# Transsynaptic entrainment of cerebellar nuclear cells by alternating currents in a frequency dependent manner

**DOI:** 10.3389/fnins.2023.1282322

**Published:** 2023-11-10

**Authors:** Qi Kang, Eric J. Lang, Mesut Sahin

**Affiliations:** ^1^Biomedical Engineering Department, New Jersey Institute of Technology, Newark, NJ, United States; ^2^Department of Neuroscience and Physiology, Neuroscience Institute, New York University Grossman School of Medicine, New York City, NY, United States

**Keywords:** transcranial AC stimulation (tACS), tDCS, tES, cerebellum, Purkinje cell synchrony, neuromodulation

## Abstract

Transcranial alternating current stimulation (tACS) is a non-invasive neuromodulation technique that is being tested clinically for treatment of a variety of neural disorders. Animal studies investigating the underlying mechanisms of tACS are scarce, and nearly absent in the cerebellum. In the present study, we applied 10–400 Hz alternating currents (AC) to the cerebellar cortex in ketamine/xylazine anesthetized rats. The spiking activity of cerebellar nuclear (CN) cells was transsynaptically entrained to the frequency of AC stimulation in an intensity and frequency-dependent manner. Interestingly, there was a tuning curve for modulation where the frequencies in the midrange (100 and 150 Hz) were more effective, although the stimulation frequency for maximum modulation differed for each CN cell with slight dependence on the stimulation amplitude. CN spikes were entrained with latencies of a few milliseconds with respect to the AC stimulation cycle. These short latencies and that the transsynaptic modulation of the CN cells can occur at such high frequencies strongly suggests that PC simple spike synchrony at millisecond time scales is the underlying mechanism for CN cell entrainment. These results show that subthreshold AC stimulation can induce such PC spike synchrony without resorting to supra-threshold pulse stimulation for precise timing. Transsynaptic entrainment of deep CN cells via cortical stimulation could help keep stimulation currents within safety limits in tACS applications, allowing development of tACS as an alternative treatment to deep cerebellar stimulation. Our results also provide a possible explanation for human trials of cerebellar stimulation where the functional impacts of tACS were frequency dependent.

## 1. Introduction

In transcranial electric stimulation (tES), electric fields that are induced in the cortex are several times higher than those in deep structures of the brain, as shown with direct measurements in humans (Guidetti et al., 2022) and animals (Asan et al., 2018). This loss of field strength with depth stands as an impediment for directly targeting deep brain structures in all types of transcranial electric stimulation paradigms, regardless of their temporal pattern (continuous, pulsed, sinusoidal or interleaved). Thus, cortical areas near the skull surface will always be advantageous to target for tES in order to keep the current levels low and thereby minimize spreading of the stimulus to adjacent areas that may confound the functional benefits. Cerebellar neuroanatomy presents a unique case where the output of the lateral cerebellar cortex projects to the CN, which then project to areas throughout much of the cerebral cortex via the thalamus. Thus, modulation of cerebellar output should be able to affect areas throughout the forebrain. A technique that can modulate the CN indirectly by entrainment of the cells in the cerebellar cortex would provide a powerful tool that can be utilized to treat a plethora of neurological disorders in which the cerebellum is involved. Indeed, it has been suggested that activation of cerebellar efferent pathways may be a more effective method for inducing plasticity in the forebrain regions than direct stimulation of them (Brunoni et al., 2011; Ferrucci et al., 2012; Bostan and Strick, 2018; Milardi et al., 2019).

The cerebellum has traditionally been thought of as critical for motor coordination. The anatomical, clinical and imaging evidence now indicate that the cerebellum also has central roles in cognition and emotion (Middleton and Strick, 2000; Stoodley and Schmahmann, 2009; Strick et al., 2009; Grimaldi and Manto, 2012), and that cerebellar dysfunction impacts these functions. For example, patients with cerebellar damage often suffer from comorbid cognitive impairments, including impaired timing, attention, memory, and language (Schmahmann, 1998; Gottwald et al., 2004). These symptoms are sometimes termed ‘dysmetria of thought’ (Schmahmann, 1998; Schmahmann, 2004), in contrast to the ‘dysmetria of movement’ classically associated with cerebellar dysfunction, and are clinically referred to as cerebellar cognitive affective syndrome (CCAS) (Pope, 2015). Consistent with these findings of cerebellar involvement in both motor and non-motor functions, projections from the CN target, via the thalamus, both motor and non-motor areas in the basal ganglia (Bostan et al., 2010, 2018; Bostan and Strick, 2010, 2018) and cortex (Bostan et al., 2018; Milardi et al., 2019). Concurrent with increased recognition of the cerebellum’s clinical importance, use of cerebellar transcranial electrical stimulation (ctES) has surged, mostly using direct currents (Ferrucci et al., 2008, 2012; Brunoni et al., 2011; Galea et al., 2011; Grimaldi et al., 2014, 2016; Hardwick & Celnik, 2014; Herzfeld et al., 2014; Priori et al., 2014). These studies show that ctES can improve motor learning, and cognitive (Ferrucci et al., 2008) and emotional processes (Ferrucci et al., 2012) in normal (Galea et al., 2011; Ferrucci et al., 2012; Hardwick and Celnik, 2014; Herzfeld et al., 2014) and brain injured individuals (for review, see Grimaldi et al., 2014, 2016; Priori et al., 2014).

Although tACS has been used in other brain areas for some time, the majority of transcranial stimulation studies in cerebellum thus far have used direct currents (DC) to perturb cerebellar networks. However, cerebellar transcranial alternating current stimulation (ctACS) has lately been garnering interest. The earliest ctACS application was an attempt less than a decade ago to demonstrate that tremor frequencies could be entrained in healthy subjects (Mehta et al., 2014). More recently, the effects of different AC frequencies (10, 50, and 300 Hz) was investigated on the excitability of the contralateral motor cortex using cerebellum-brain inhibition (CBI) (Naro et al., 2016, 2017). CBI is mediated by Purkinje cell (PC) projections from the cerebellar cortex that are inhibitory to the CN cells, many of which, in turn, provide excitatory projections to the motor cortex via the thalamus. Five minutes of 50 Hz ctACS as a conditioning stimulus caused weakening of CBI, quantified by short-lasting elevations in the excitability of the contralateral M1. Interestingly, the 300 Hz stimulation had the opposite effect on CBI and the 10 Hz ctACS was ineffective, suggesting a frequency dependency of ctACS.

Animal studies investigating the underlying mechanisms of ctACS, and ctES in general, are scarce. Electric fields required to modulate Purkinje cell spiking activity was studied in an *in vitro* turtle cerebellum preparation (Chan and Nicholson, 1986). In 2020, Asan et al. reported the first *in vivo* study that demonstrated entrainment of the PC simple spikes (SS) in the rat cerebellar cortex by AC stimulation in an amplitude, frequency, and direction dependent manner (Asan et al., 2020). Phase-locking of SS to AC cycle became stronger as the frequency was increased from 2 Hz to 40 Hz. Further increasing the stimulation frequency to 100 Hz, beyond the spontaneous frequency of the cells, led to spikes skipping the AC cycles while maintaining the phase locking.

The principle mechanism that we aim to leverage here is PC-SS synchrony. Several dozens of PCs converge on each CN cell with inhibitory (GABAergic) connections (Wu and Raman, 2017). Transient synchrony among the SS of those projecting PCs have been shown as a potential mechanism for modulation of the CN cells (Person and Raman, 2011, 2012). Thus, entrainment of a group of PC-SSs around the stimulation electrode by the same AC waveform should lead to synchrony and thereby modulation of the CN cell spikes at the stimulation frequency. The results suggest that CN cell modulation via stimulation of the cerebellar cortex is feasible in an intensity and frequency dependent manner, and that the most effective range of AC frequencies varies between CN cells.

## 2. Methods

### 2.1. Animal surgery

Experiments were performed in accordance with the National Institute of Health *Guide for the Care and Use of Laboratory Animals*. All procedures were approved and performed in accordance to the guidelines of the Institutional Animal Care and Use Committee, Rutgers University, Newark, NJ. Eleven Sprague Dawley rats (200–350 g, male, Charles River) were used in this study. Animals were first anesthetized with 5% isoflurane in an induction chamber, and then moved to a stereotaxic frame. The animal was switched to ketamine/xylazine mixture (80 mg/kg and 8 mg/kg, IP) during the course of surgery and data collection. Additional doses of ketamine (15 mg/kg, IP) were injected as needed such that the pedal reflexes were absent. Body temperature was measured with a rectal probe and regulated with a heating pad under the animal (ATC 1000, WPI, Sarasota, FL). Blood oxygen level was monitored with a pulse oximeter attached to the hind paw. The hair over the head was shaved and a midline skin incision was made to expose the skull over the cerebellum. A craniotomy hole was opened for insertion of the CN recording electrode at AP = −11.5 mm on the midline and extended laterally to the right side for 2 mm. For stimulation of the cerebellar cortex, the skull over the right side of the posterior cerebellum and the vermis was opened with rongeurs and the dura was kept intact and moist with normal saline.

### 2.2. Stimulation of cerebellar cortex with AC

**Figure 1:**
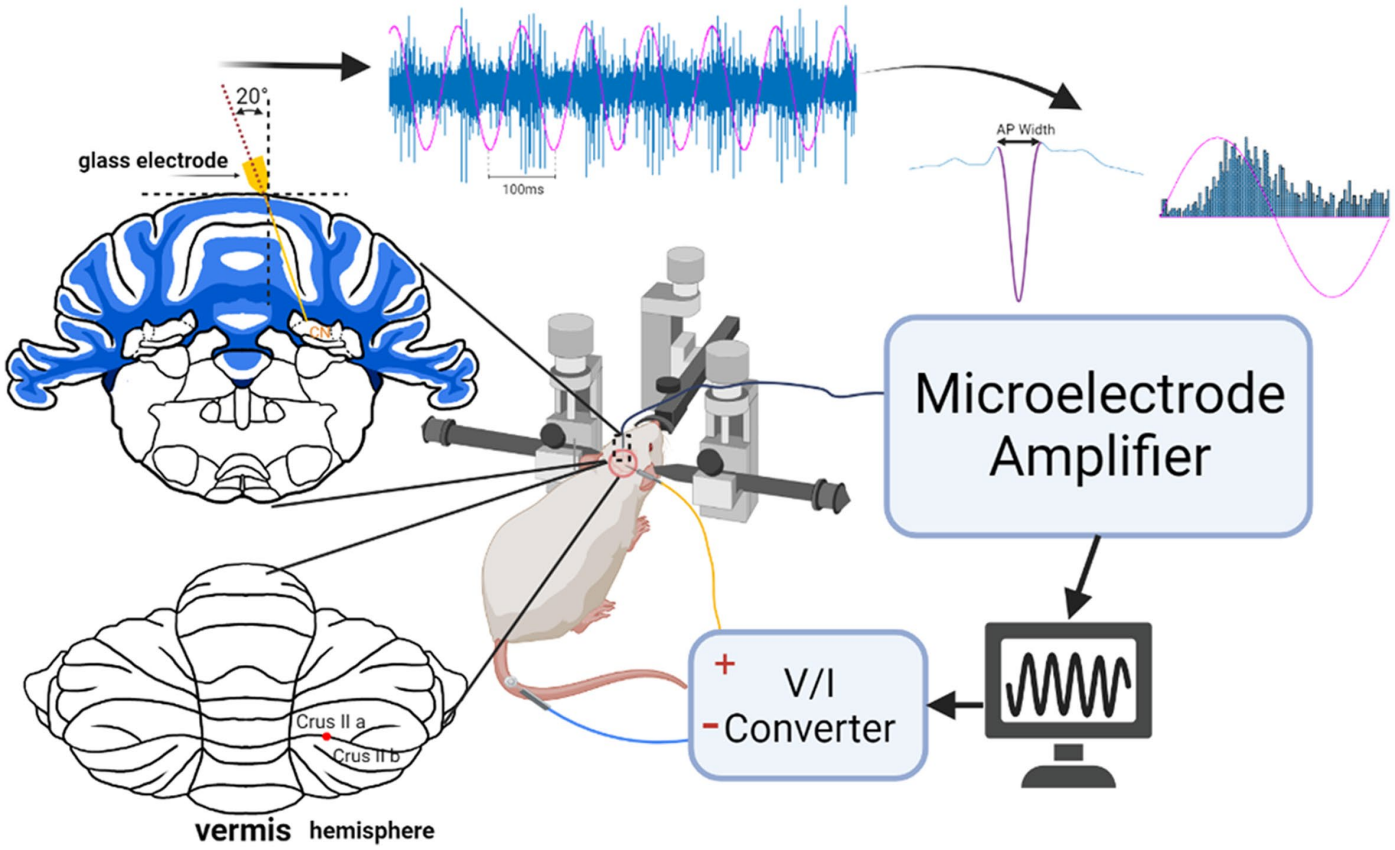
Experimental setup for stimulation of the cerebellar cortex and recording CN activity. The coronal section on top left shows the penetration angle of the recording microelectrode through a craniotomy on the dorsal side. The posterior view of the cerebellum depicts where the cortex was stimulated at the border of crus IIa and IIb about 2 mm from the paravermal vein with a circular Ag/AgCl electrode (flat end of a wire). The raw signal is an example of CN spike entrainment and the associated histogram. AC waveform (magenta) is superimposed to show the stimulation phase.

Stimulus waveforms were generated in Matlab (Mathworks, MA), sent out through a data acquisition card (PCI-6071, National Instruments, Austin, TX), and passed through a V/I converter (Linear Current Isolator, Caputron, Hillsborough, NJ) before being applied to the cerebellar surface via a stimulus electrode (Figure 1). The stimulus electrode consisted of a silver wire (0.5 mm diameter) whose tip was sanded (220 grit paper), to obtain a flat circular footprint, and coated with AgCl, by immersing it in 1.84% sodium hypochlorite solution overnight. The circular tip was pressed against the dura to deliver the AC stimulation at different points across the cerebellar cortex. A disposable electromyogram electrode was attached to the tail as the return electrode for the stimulus current. The 1 kHz AC impedance between the two electrodes was typically around 12 kΩ. Any fluids accumulating around the stimulus electrode were removed as needed using Q-tip cotton applicators to minimize the spread of current laterally over the dura.

### 2.3. Electrophysiological data collection

**Figure 2:**
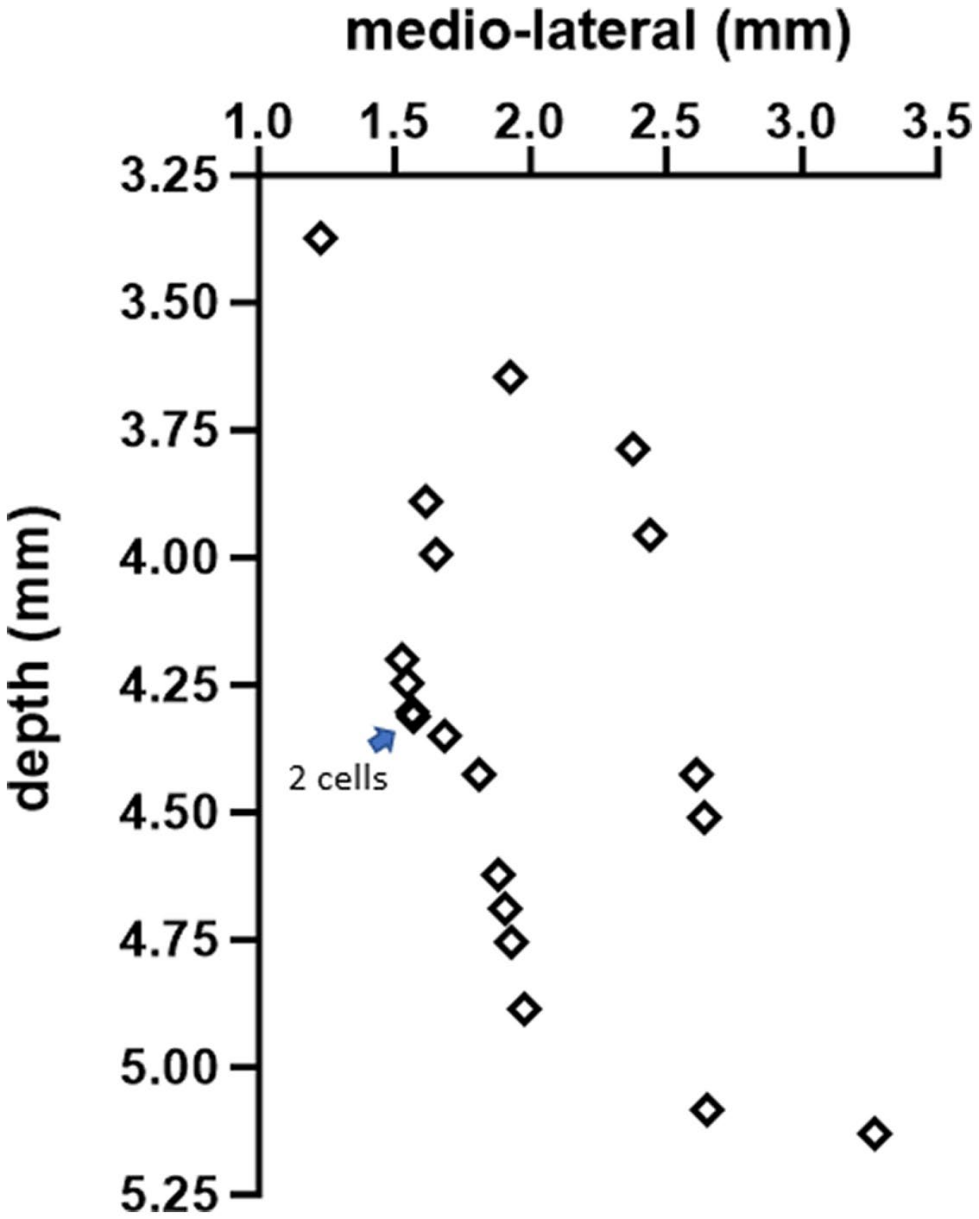
The estimated positions of all 20 cells, based on stereotaxic coordinates, in the coronal plane at AP = −2.5 mm from the interaural line.

**Figure 3:**
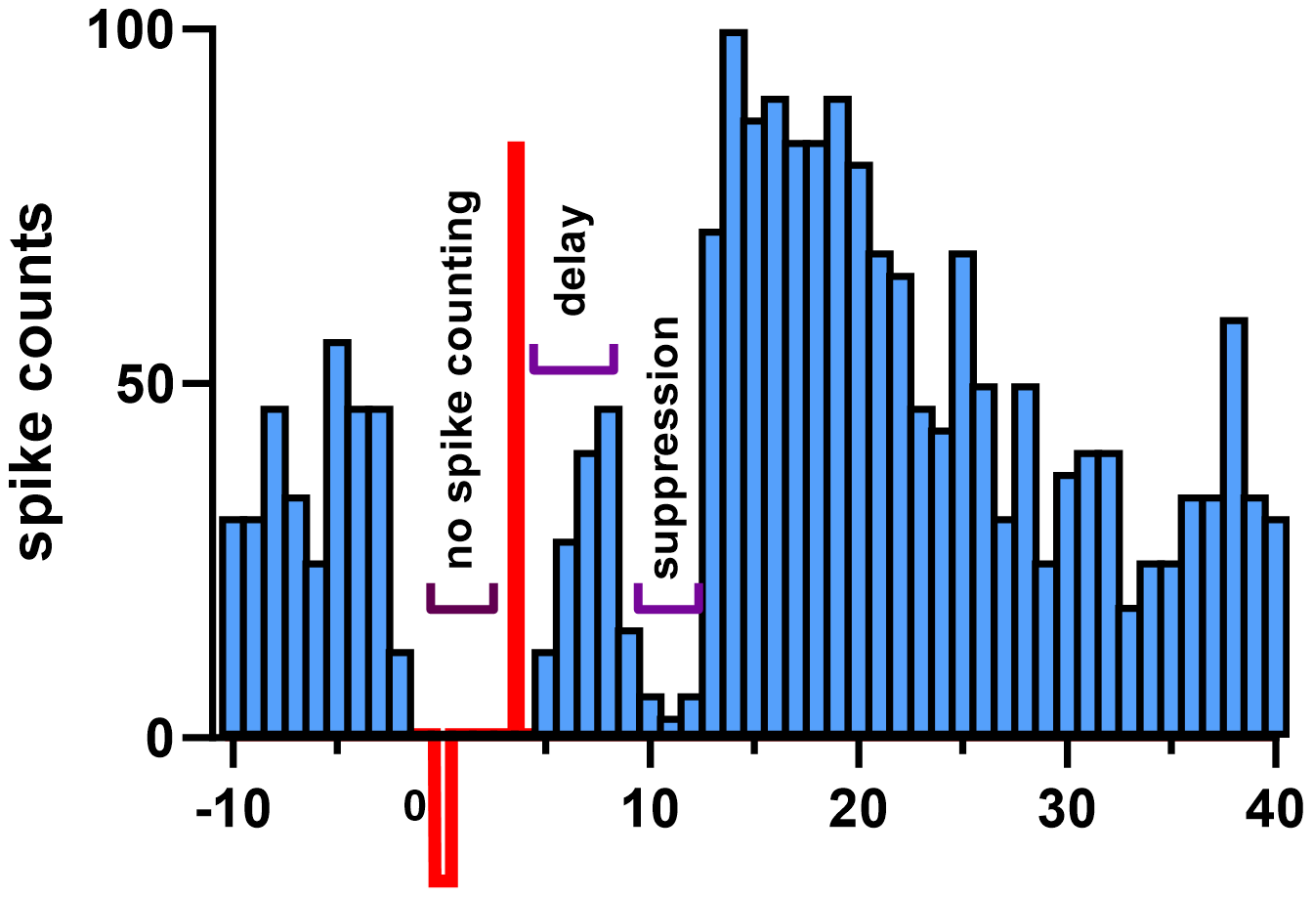
Demonstration of synaptic connectivity from the stimulated cortical area to a CN cell. Stimulus triggered histogram of CN spikes shows inhibition. One hundred biphasic current pulses (200 μs anodic pulse, preceded by a 2 ms gap and a 800 μs cathodic phase for charge balancing) were applied through the same cortical electrode used for AC modulation. The CN spikes are suppressed for a few ms after a delay that can account for the propagation time from the cortex to CN. In most cases, the anodic simulation was more effective than the cathodic one to produce such inhibition. Notice that the spike count increases above the baseline after the inhibition period. Spikes were not counted during the biphasic stimulus waveform due to artifact.

A glass micropipette (3–4 MΩ) filled with normal saline was inserted through the cranial hole on top at a lateral angle of 20° with the vertical axis and a rostrocaudal position of −2.5 mm from the interaural line to target the interpositus nucleus (IPN). The estimated mediolateral positions and the depths of the recorded cell positions are shown in Figure 2, based on the stereotaxic coordinates. Signals were filtered at 300 Hz–5 kHz and amplified (Model 2200, A-M Systems, Carlsborg, WA) by a gain of 1,000 or 10,000, sampled at 100 kHz onto the computer via a data acquisition board (PCI-6071), and analyzed during the experiment. Neural signals were monitored simultaneously on an oscilloscope and an audio speaker. Each trial consisted of a 10 s long baseline recording followed by a 20 s long stimulation at frequencies ranging from 10 Hz to 400 Hz to investigate entrainment of the CN activity through cortex stimulation. The stimulus current amplitudes were varied from 10 μA to 60 μA in steps of 10 μA. All current amplitudes are given as the peak of the sinusoidal waveform. Before collecting AC data, an anodic- or cathodic-first biphasic and charge-balanced current pulse (20 Hz, 200–300 μA, duration 200 μs, 2 ms gap between phases to minimize the hyperpolarizing effect of the first pulse) was applied through the stimulation electrode on the cerebellar cortex with respect to another similar electrode at ~1 mm lateral distance (bipolar stimulation) to ensure that the PCs underneath the stimulation electrode were projecting to the CN cell that was recorded. The paravermal area in crus II (~2 mm from the paravermal vein, Figure 1) was found to produce the strongest CN inhibition with current pulses and the lowest thresholds for CN modulation with AC. The connectivity was confirmed for each of the 20 cells from 10 animals (1 cell in each of 3 rats, 2 cells in each of 7 rats, and 3 cells in 1 rat) with a spike-triggered histogram that showed a period of silence or significantly decreased activity following the stimulus pulse (Figure 3). In 13 out of 20 cells, the AC stimulation was also superimposed on a positive or negative DC current with different amplitudes to observe the combined effect (AC + DC) on the CN spike entrainment as well as the mean firing rates.

### 2.4. Spike analysis

All data processing was performed in Matlab. Raw signals were band-pass filtered (300–5,000 Hz or 500–5,000 Hz depending on the stimulus frequency) and the negative spikes were detected by thresholding. Then, scatter plots were produced using the first two principal components of the detected action potential waveforms within a 1.5 ms window around its negative peak. The user, then, manually selected a cluster of spikes that putatively belonged to a single cell. Time points of the selected spikes were plotted with respect to the AC stimulus phase as a histogram. The phase lock value (PLV) was defined (Krause et al., 2019) as PLV = abs(mean(e^iθ^)), where the θ are the phase angles of individual spikes with respect to the AC cycle, and used as an index of CN modulation or entrainment. The time lag of the spike entrainment was found from the angle of the PLV vector. The modulation levels that are detectable by PLV depend on the duration of the signal (Mc Laughlin et al., 2022). In order to determine the smallest PLV level that can detect modulation above the chance level for the length of the episodes we have, the PLVs were computed from all the baseline recordings available in a cell (~120 10-s epochs were combined in pairs to make ~60 20-s epochs matched in duration to the stimulation episodes). Then, the PLV threshold corresponding to the probability of 5% error of false positive was calculated from the distribution for each cell. The average of all threshold PLVs from 20 cells was 0.057 (min = 0.042 and max = 0.088). Based on this analysis, we considered any PLV above 0.088 as detectable modulation and used 0.15 in some cases as a more conservative value.

The interspike intervals (ISIs) were computed for the baseline and stimulation periods and they were compared using Kolmogorov–Smirnov (KS) tests for the probability distributions, and two-tailed paired-*t* tests for the means. After-effects of the stimulus were not analyzed in this study because the short stimulus durations that were used were not expected to produce any plastic effects.

## 3. Results

### 3.1. Measurement of cerebellar volume conductance

Electrical stimulation applied to the cerebellar cortex can have both direct effects on the CN cells and transsynaptic effects through PC modulation. In order to show that the electric fields (e-fields) directly induced on the CN cells are relatively much smaller than the e-fields induced on the PCs of the cortex, we employed both direct measurements of the e-fields in the cerebellum and the analytical method of estimation. The conductivity of the cerebellum as a volume conductor needs to be known to solve the analytical equations of the e-field. To this end, a train of current pulses was applied to the stimulating electrode while the electrode back voltage was recorded. The impedance of the electrode-tissue interface was excluded from the measurements by identifying the capacitive charging component of the waveform and taking only the initial sharp edge of the voltage transition into account. Then, using the voltage equation at the surface (*V=I/4σa*, where *I* is current and *a =* 0.5 mm is the electrode radius) derived for a disk electrode on the surface of a semi-infinite medium (Wiley and Webster, 1982), the lumpsum equivalent conductivity (*σ*) for the cerebellum as a volume conductor was found to be 0.42 S/m.

### 3.2. Predicted and measured electric fields in the cerebellum

The voltage distribution inside a semi-infinite volume conductor for a disk electrode was analytically solved by Wiley and Webster (1982). One can find the electric field by differentiating their voltage equation given in polar coordinates. The vertical e-field (*E = I /* [*2πσ*(*a^2^ + z^2^*)]), found by differentiating the Wiley’s equation in the z direction, was estimated to be around 150 mV/mm at the PC layer (depth of ~*z* = 300 μm including dura) for a 60 μA current, electrode diameter of a = 0.5 mm, and our measured conductivity of 0.42 S/m. This equation predicts that the e-field strength is reduced to 1.12 mV/mm (by a factor of ~123 from the PC layer) at a depth corresponding to about the middle of CN (*z* = 4.5 mm) assuming a homogenous and isotropic medium.

We also measured the e-fields experimentally in the CN using 10 Hz, 100 Hz, and 1 kHz sinusoidal currents as well as rectangular pulses (Asan et al., 2018). A recording electrode (1.5 MΩ glass micropipette) was advanced in steps of 200 μm into the CN from an insertion point next to the simulating electrode that was located ~2–3 mm lateral from the midline and near the crus IIa - crus IIb border, which we found as the most effective region on the cortex for CN modulation. The measured e-field at a depth of 4.5 mm was 1.74 mV/mm, 1.5 mV/mm, and 1.44 mV/mm with the three sinusoidal currents respectively, and 1.5 mV/mm with the rectangular pulses for the largest (60 μA) stimulation current used. These e-fields in the CN were in the same ballpark with the value predicted by the theoretical calculations (1.12 mV/mm) above. It should be mentioned, however, that the decline in the e-field by depth may not be so steep depending on where in the cerebellum the measurements are made with respect to the walls of folia (Avlar et al., 2023), in part because of higher conductivities along the axon tracts (anisotropy).

### 3.3. Verifying PC-CN connectivity by cortical stimulation

In order to verify that we stimulated a cortical area that projected to the CN cell being recorded, we applied brief shocks with a bipolar electrode to that area and looked for responses whose sign and latency indicated a direct synaptic connection. In Figure 3, the mean delay from the stimulus pulse to the start of inhibition was 2.31 ± 0.92 ms (13 cells). In the remaining 7 cells, the start of the inhibition was not possible to determine due to stimulus artifact, however, the end of inhibition period was clearly visible. The average duration of inhibition was 2.56 ± 2.03 ms (range 0.4 ms to 9.0 ms, 13 cells). IPSC decay times vary dramatically from ~2 ms for the large CN cells to ~25 ms for some of the small nucleo-olivary CN cells in mice (Najac and Raman, 2015). Thus, the brevity of the inhibition we observed strongly suggests that CN cells of this study mostly belong to the class of large non-GABAergic CN cells, which include the cells that project to the thalamus.

### 3.4. Spike histograms were mostly monomodal

**Figure 4:**
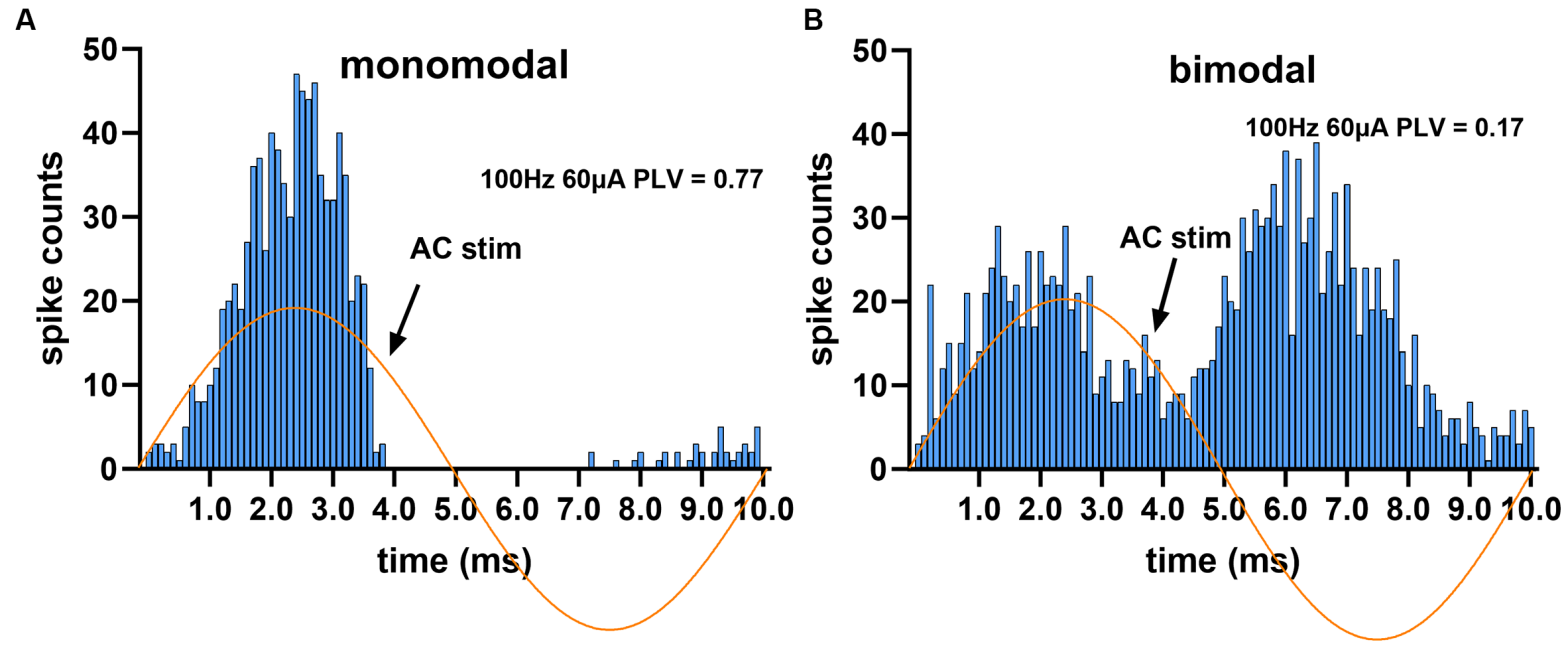
Spike timing histograms from two different cells, triggered off of the AC cycle. AC stimulus amplitude was set to the highest (60 μA at 100 Hz) to maximize entrainment. **(A)** Monomodal distribution with a single peak and a complete suppression during most of the negative cycle. **(B)** The second peak during the negative cycle gives the appearance of a bimodal distribution.

The responses of CN neurons to AC stimulation were visualized by plotting histograms triggered off of the start of the AC cycle. Most of the histograms had a mono-modal distribution as in Figure 4A, but in very few cases the distribution was bimodal (Figure 4B, right panel). The PLV statistic was used to quantify the modulation apparent in the histograms. A PLV value of zero indicates that spikes distributed uniformly across the stimulus cycle, and PLV produces a maximum value of 1 when the entrainment is perfect, i.e., all spikes occur with the same exact phase lag in the cycle. It produced a maximum value of π/4 in our simulations if the spike histogram had the appearance of the positive half cycle of a sinusoidal, similar to the one in Figure 4A. However, PLV yields a small value for symmetrical distributions even if the modulation is substantial, such as the one that resembles a rectified sinusoidal waveform as in Figure 4B, because of spikes canceling each other’s contributions to the mean that are 180° apart around the unit circle. The bimodal cases were fewer in number and did not affect the overall conclusions (13% of all trials). The elevated spike probability in negative AC cycle of these bimodal histograms were possibly produced by the projecting PCs that were located on the periphery of the stimulated cortical area and their somatodendritic axis was oriented in the electrical field in the opposite direction.

### 3.5. CN spike activity modulates with a short latency with respect to AC peak

**Figure 5:**
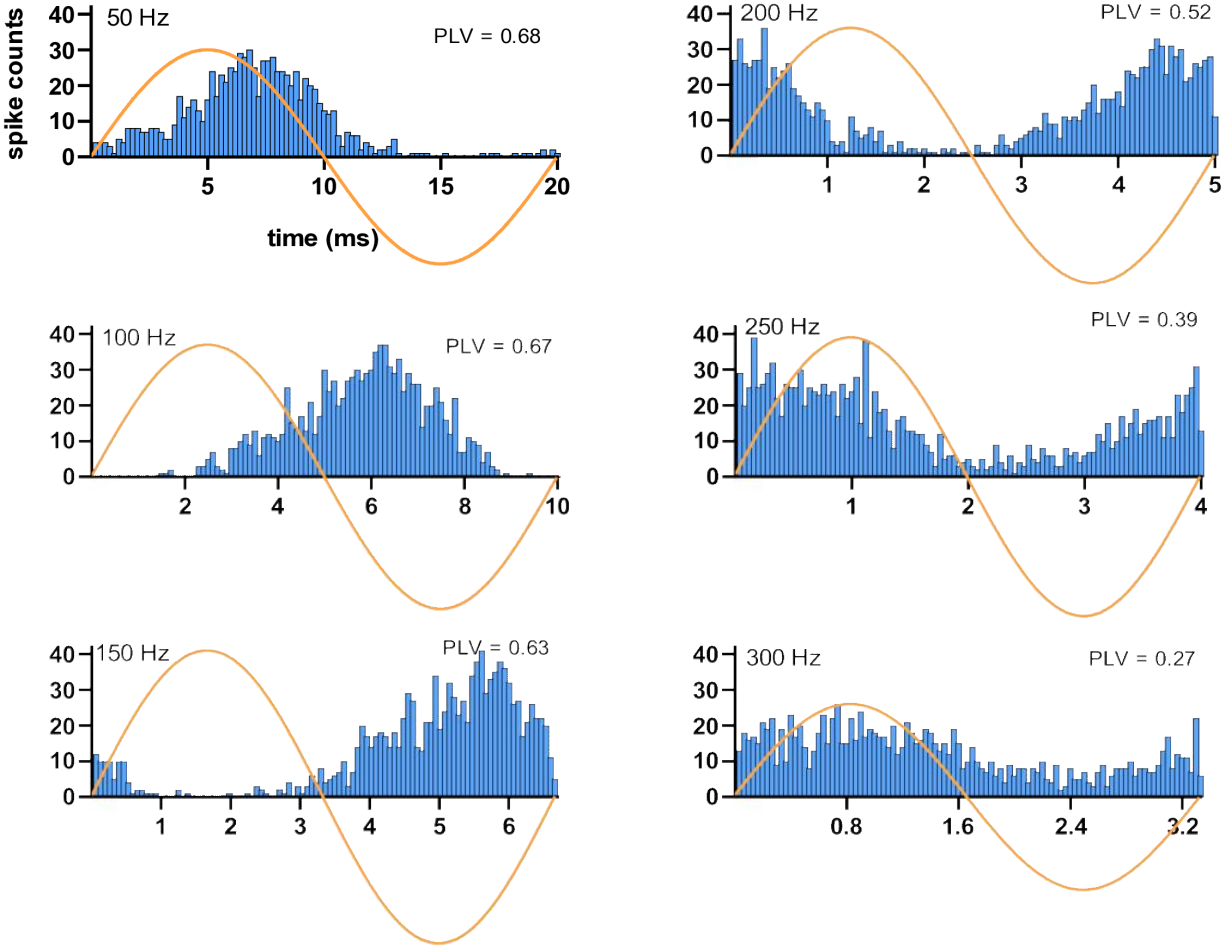
Spiking timing histograms for a CN cell at stimulation frequencies ranging from 50 Hz to 300 Hz. Note the change in the latency of the modulation with respect to the AC cycle. Stimulus amplitude is 40 μA. PLVs are also indicated in each case.

Histograms triggered off the start of the AC cycle showed entrainment of the CN activity at the AC frequency (Figure 5). At 50 Hz, the histogram shows a peak that lags the peak of the sine wave by approximately 1/8 of a cycle (2–3 ms) and that is followed by a suppression of activity during the negative half of the AC cycle. As the stimulus frequency is increased from 100 Hz to 250 Hz, CN activity shows an increasing phase lag, until at 250 Hz the lag is so great that the CN activity appears to lead the AC cycle and at 300 Hz has an essentially in-phase relationship albeit with a much weaker modulation amplitude. At the other end of the frequency range (10 Hz), the histograms had such wider distributions that made it difficult to find a clear peak for delay measurements.

Considering this frequency dependence, time lags were measured using the data at 100 Hz stimulation where entrainment was strongest and the cycle length was long enough to find the histogram peak in the same AC cycle. The average and median time lags were 4.3 ± 2.7 ms and 5.4 ms (31 trials, 14 cells), respectively. Overall, the modulation lags measured from the histograms suggest that the CN cells generated a spike with short latencies following the AC waveform peak. Furthermore, the short delays in CN inhibition following pulse stimulation of the cortex (see “PC-CN Connectivity” above) are in agreement with having such short lags in the modulation histograms.

### 3.6. No change in mean firing frequency

**Figure 6:**
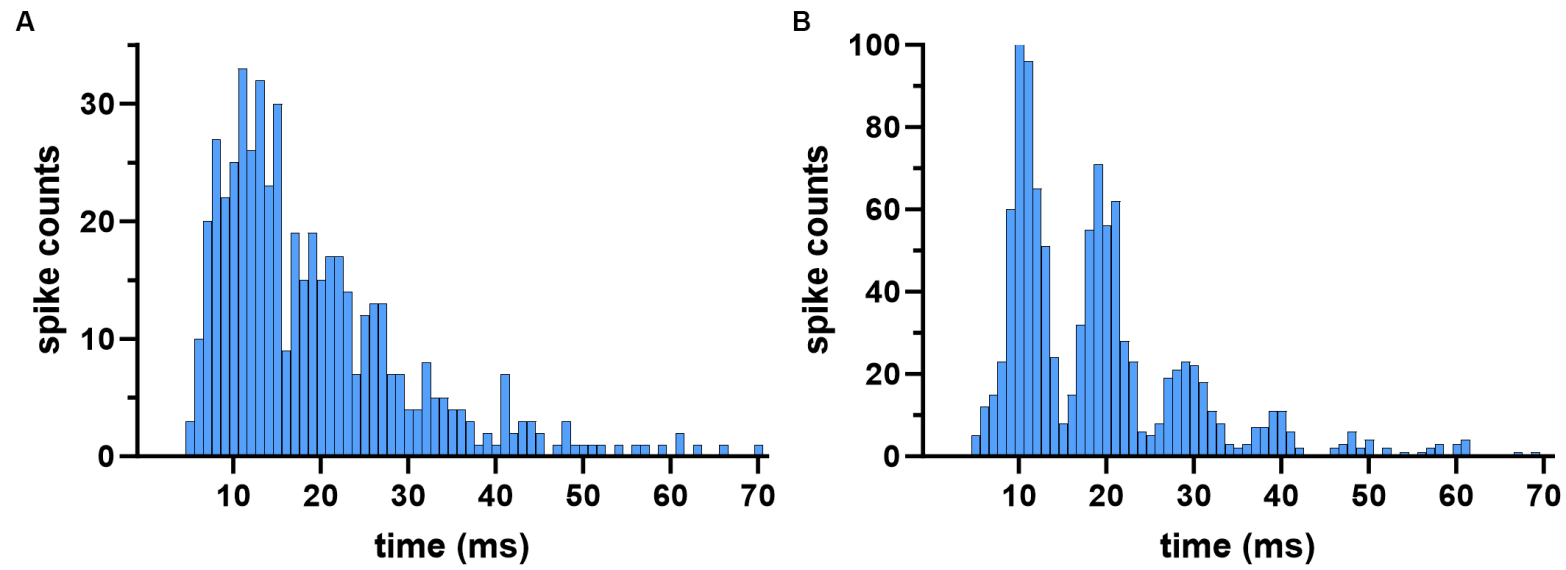
Inter-spike-interval histograms during the baseline **(A)** and stimulation **(B)** periods in a cell modulated at 100 Hz. Histogram peaks occur at multiples of the AC cycle (10 ms) during stimulation indicating modulation at subharmonic frequencies. Subharmonic peaks emerge when the spikes start skipping one or more AC cycles, which tended to occur when the stimulus frequency is larger than the cell’s spontaneous firing frequency.

In most cases, the envelope of the ISI distribution of a cell did not change substantially but assumed a multi-peak appearance during stimulation (Figure 6). Particularly in cases with strong modulation, the ISI histograms had a clear peak corresponding to the AC stimulus cycle, but also at multiples of it, showing entrainment of the spikes at subharmonic frequencies (Figure 6B). This multi-peak histogram pattern is an indication that the consecutive spikes are skipping one or more AC cycles when the stimulation frequency is higher than the spontaneous firing frequency of the cell. This raises the question of whether the average firing frequency of the cell is modified by the stimulation relative to its baseline rate.

In 427 out of 492 trials (of all stimulation amplitudes and frequencies in 20 cells) where the modulation was clearly above the noise level (PLV > 0.15) and the stimulation frequency was higher than the spontaneous firing rate of the cell, no significant change was found in the mean firing frequency of the cells from the baseline (*p* = 0.2, two-tailed Student’s t). However, the K-S test indicated that the ISI distributions were altered (*p* < 0.05) in the same trials. In 236 (55%) trials out of these 427, the spiking frequency was decreased (from 46.8 ± 18.6 spikes/s to 37.6 ± 20 spikes/s) and in the remaining 191 (45%) trials increased (from 44.4 ± 20 spikes/s to 51.8 ± 19.4 spikes/s), although the stimulation frequency was higher than the spontaneous firing rate in all these cases. Person et al. also observed an increase in the firing of some CN cells and a decrease in others under synchronized PC inhibition (Person and Raman, 2011). The changes in the firing rates must be due to spontaneous variations, rather than a result of stimulation, although the baseline recordings for comparison were immediately preceding the stimulation interval. Thus, it seems that spontaneous variations of the cells were strong enough to obscure any alterations induced on the mean firing rates by the AC stimulation in these 20-s stimulation episodes.

### 3.7. Frequency tuning of modulation

**Figure 7:**
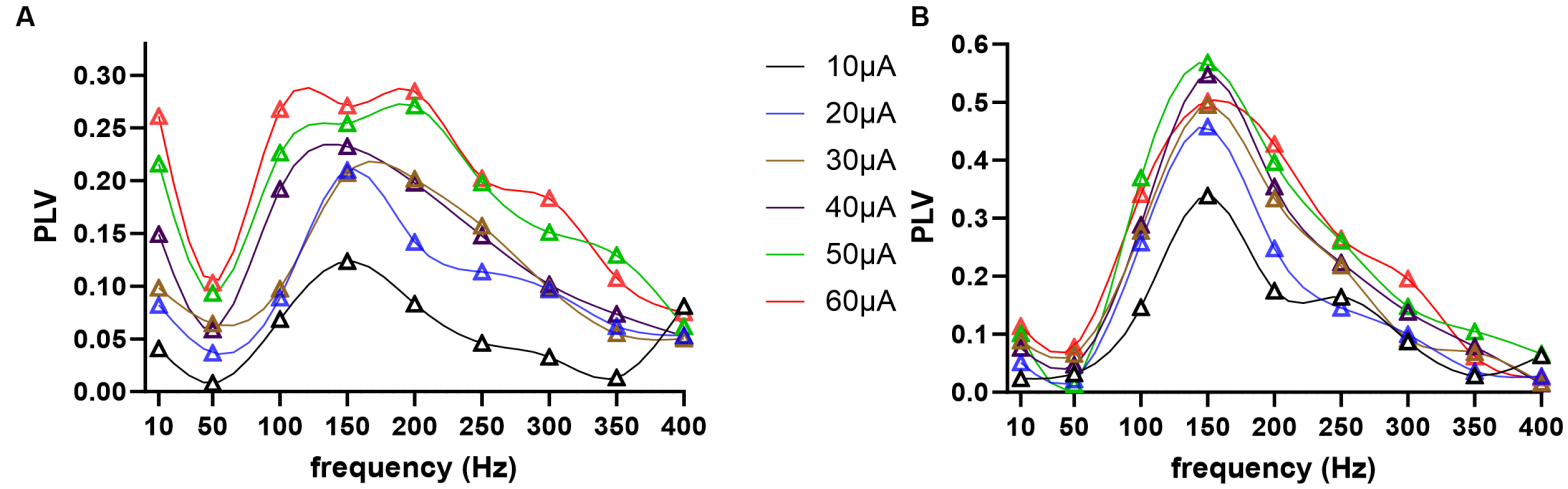
Modulation index, phase lock value (PLV), as a function of frequency and stimulus amplitude, coded in different colors, for two sample cells in **(A,B)**.

All cells were entrained by the AC stimulation in an amplitude and frequency dependent manner, as shown in two sample cells in Figure 7. The modulation index as a function of frequency revealed a tuning curve where a range of mid-frequencies were most effective. In the first cell (Figure 7A), maximum entrainment occurred at 150 Hz for 10–40 μA stimulation, and moved to 200 Hz for 50 μA. In this particular cell, there was an increase in modulation at 10 Hz for larger stimulation amplitudes. However, this was a rare case, rather than the norm, and it was perhaps due to direct modulation of the CN cell through volume conduction of the stimulation current, and not due to transsynaptic modulation. In the second cell (Figure 7B), the PLV plots were more consistent for different stimulation amplitudes, perhaps because the entrainment was stronger and clearly above the noise level.

In general, the peak modulation levels increased as a function of stimulus amplitude as expected, but also varied between the cells for the same current. However, this may be a matter of finding the best spot for stimulation on the cortex that is well connected to the recorded CN cell, rather than a reflection of some electrophysiological characteristics of the cell related to how effectively the cell can be modulated transsynaptically. The peak modulation frequency also varied between cells, ranging from 50 to 250 Hz. For instance, with 30 μA AC stimuli, 5 cells had their maximal PLV at 50 Hz, 7 cells at 100 Hz, 6 cells at 150 Hz, 2 cells at 200 Hz, and 1 cell at 250 Hz.

**Figure 8:**
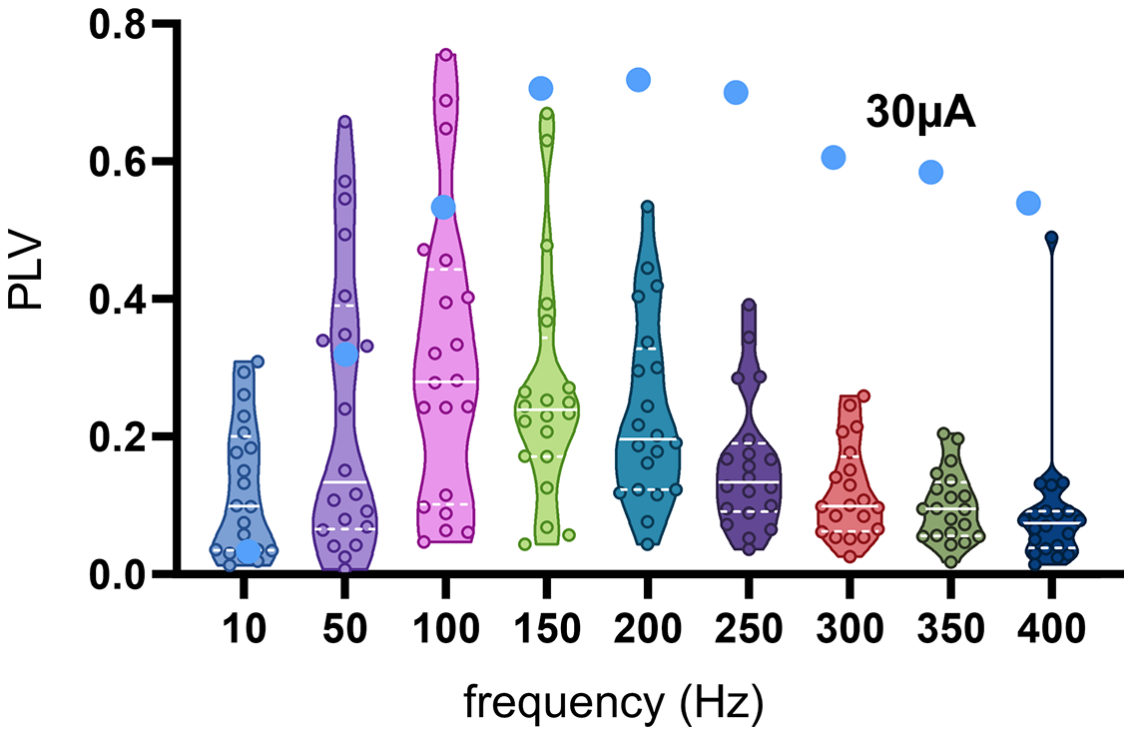
PLV as a function of frequency from all cells at 30 μA stimulation. Small circles are actual data points from 19 cells, the horizontal dash lines are the medians, and the dotted lines are the quartiles. The blue filled circles belong to the 20th cell, which had a different trend than the rest with substantial modulation even at the higher end of the frequency range.

The frequency dependence of the modulation amplitude for the entire population of recorded CN cells is shown in Figure 8, where the PLV of each cell is plotted for each tested frequency. In order to minimize the risk of direct modulation of the CN by the AC stimulus, PLVs plotted in Figure 8 were in response to 30 μA AC stimulation, which was large enough to produce significant transsynaptic modulation (e.g., Figure 7, brown curve) and further increases of stimulus intensity produced only minimal increases in PLV. In general, 10 Hz was very inefficient for modulation. The PLV decreased toward higher frequencies after its peak, except in one cell that maintained a PLV of 0.5 up to 400 Hz (filled circles).

### 3.8. Correlating the modulation type to cellular electrophysiology

A positive correlation was found between the action potential durations from all cells and their peak PLV frequencies (*r* = 0.35, *p* = 0.0002). The true correlation, however, may be higher, as the band-pass filter (fc = 300 Hz or 500 Hz to 5 kHz) that we had to use to eliminate the stimulation artifacts from the data may have also filtered out some of the cell-to-cell differences in spike durations. The average spike durations during baseline recordings was 0.62 ± 0.18 ms, which is closer to that of the large cells than the nucleo-olivary cells in mice (half-width is 0.3 ms vs. 1.05 ms, respectively, in Najac and Raman, 2015), particularly as we used the full amplitude of action potentials to measure the spike widths (inset in Figure 1). Uusisaari and Knöpfel set the 0.6 ms half-width duration as the upper threshold to differentiate the large non-GABAergic cells from the small ones and the GABAergic cells also, which had an average half-width of 0.99 ms (Uusisaari et al., 2007; Uusisaari and Knopfel, 2011). Thus, the CN cells of this study were estimated to include mostly the large non-GABAergic cells, and imply that they show significant heterogeneity in their modulation characteristics, such as the PLV peak frequency.

The highly sporadic nature of the CN spike train patterns (coefficient of variation-CV of ISI is 0.62 ± 0.5) made it difficult to classify the cells based on their mean firing frequencies. For instance, the average spontaneous firing frequency was 51.7 ± 19.6 Hz (*N* = 1,754 trials from 20 cells). The mode of firing frequency (i.e., the reciprocal of the modal interspike interval in the baseline period preceding each trial), however, was much higher (107 ± 47 Hz), suggesting short episodes of high frequency bursts in the baseline. It should be mentioned, however, that imperfections in isolating the spikes from a single cell using the principal components method can lead to contributions from nearby cells and thereby cause miscalculations on spike firing rates and durations.

### 3.9. AC+DC stimulation

**Figure 9:**
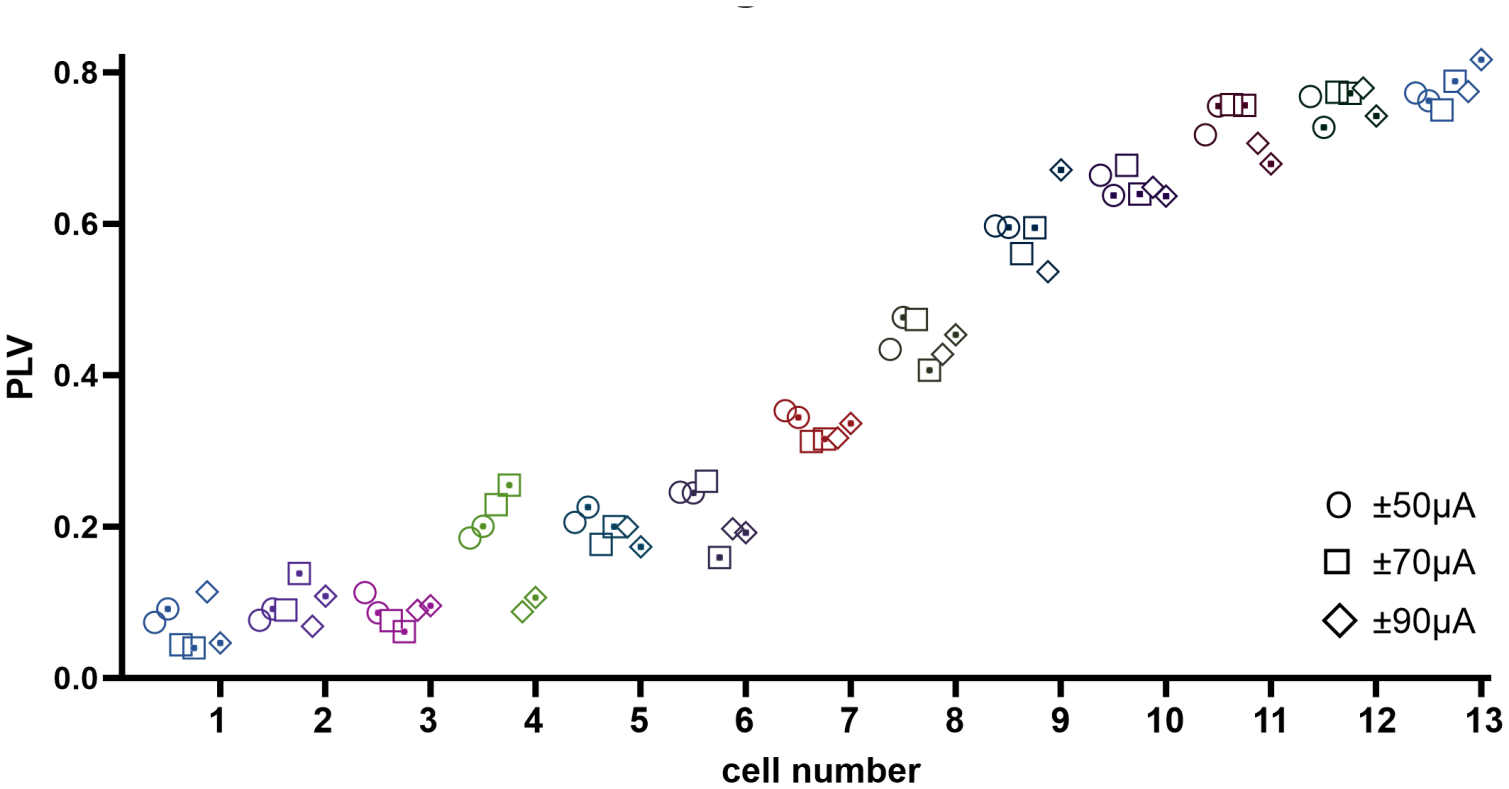
PLV measures from 13 cells (coded in different shapes with dot (negative DC) and without dot (positive DC) inside) at ±50, ±70, and ± 90 μA DC (from left to right respectively) superimposed on 100 Hz 50 μA AC stimulation. The cells are ordered with increasing PLV values left to right for appearance only. Overall, DC component has no significant effect on the PLV within each cell.

Finally, we were interested to know if altering the PC baseline firing rates with a DC component would influence the level of CN modulation by the AC stimulation. Thus, entrainment of CN spikes was tested with a DC offset superimposed on the AC stimulus waveform in 160 trials in 13 cells from 8 animals. The DC offset was set to ±50, ±70, and ± 90 μA to which a 100 Hz, 50 μA peak amp AC signal was added. Interestingly, the PLV was observed to change only as a function of the AC stimulus frequency and amplitude (data not shown) as in other trials, but it was independent of DC amplitude and/or polarity (Figure 9), i.e., no significant difference in PLV was found in all cases due to DC (ANOVA, *F* = 0.01, *p* = 0.98). These results lead us to two significant conclusions. One, the DC stimulation of the cerebellar cortex, which was shown to alter the PC spike frequencies almost immediately (Asan et al., 2020), does not affect the modulation of the CN cells induced by the AC component of the stimulus. This suggests that altering the PC baseline firing rate may not necessarily predispose the PCs for stronger or weaker synchronization of the simple spikes, at least not at the current levels that were tested here. Two, the direct effects of the cerebellar cortex stimulation on the CN cells through volume conduction was not significant enough to alter the results of transsynaptic modulation from the AC stimulation of the cortex.

## 4. Discussion

Our goal in this study was to investigate if the CN cells could be entrained transsynaptically by alternating currents applied to the cerebellar cortex. We employed electric stimulation of the cortex with rectangular pulses (to show inhibition of CN cells by PC activation) to ensure that the cortical area being stimulated projected to the CN cell that is recorded. Moreover, the cortical location with the lowest threshold for modulation was searched for, and the AC amplitudes were kept low in order to minimize the direct effects on the CN cells through volume conduction of the current.

In our data, all 20 cells estimated to be in the CN by stereotaxic coordinates and confirmed to be projected by the cortical cells showed modulation by an AC stimulus in an amplitude and frequency dependent manner. In contrast, in the pre- and postcentral gyri of awake nonhuman primates, only 10–20% of cells were responsive to 10 Hz tACS (Johnson et al., 2020). Responsiveness of neurons to tACS or the lack of it was ascribed to differences in their morphology, orientation in the electric field, and dynamic biophysical properties. In our recent study, lower frequencies (0.5–20 Hz) and larger currents were tested to directly modulate the cortical and nuclear cells in the rat cerebellum (Avlar et al., 2023); where essentially all cells were responsive to the stimulus, though to different levels and with phase delays that varied with their locations with respect to the stimulus electrode. An advantage of transsynaptic modulation of CN cells is that the electric field direction only matters at the cortical level for the cells that are under the direct effect of the current. In the cerebellar cortex, especially atop the folia, the PCs, the cortical cells that project to the CN, have a well-known orientation and position with respect to the induced electric fields. Nevertheless, the variations in the electrophysiological properties of the PCs across the cortex in different zebrin bands (Xiao et al., 2014; Zhou et al., 2014; Tang et al., 2017) and lobes, as well as the PC to CN cell projection maps (Sugihara and Shinoda, 2007; Chung et al., 2009; Sugihara, 2011), may need to be considered for more targeted functional results.

### 4.1. Frequency tuning

The decline in the PLV curve below the peak frequency reflects an interesting phenomenon (Figures 7, 8). The reason behind this decline could not be at the PC level, since the PCs were entrained at frequencies even as low as 2 Hz (Asan et al., 2020), or even 0.5 Hz (Avlar et al., 2023) with cortical stimulation. A more likely mechanism is that SS synchrony among the PCs that are under the influence of the stimulation current would become much weaker at the lower frequencies since the spikes are now distributed over a much longer time period. If we consider one half the AC cycle as the duration within which the PC spike timings are rearranged to synchronize, this time interval is 10 ms to 2 ms for frequencies from 50 Hz to 250 Hz, where the entrainment is the strongest. This interval agrees with most reports on PC SS synchrony that found spikes occurring within ~4 ms of each another (Shin and De Schutter, 2006; Heck et al., 2007; de Solages et al., 2008; Wise et al., 2010). The half cycle for 10 Hz (50 ms), however, is relatively much longer to have a useful level of spike coincidence between the PCs being modulated. Additional support for the SS synchrony being the mechanism for CN entrainment is provided by the AC + DC data where the DC offset in the waveform did not alter the CN modulation levels induced by the AC component alone, despite the fact that DC offset is expected to affect the PC spike rates (Asan et al., 2020). This suggests that using electric currents with alternating waveforms is an efficient way of transiently increasing PC spike synchrony for CN entrainment, and the average PC firing rate does not predispose the PC synchrony to strengthen or weaken the CN entrainment, not at least when the AC and DC stimulation components are in the same amplitude range.

The negative slopes at the higher frequencies of the PLV curves also need an explanation. An excellent study by Lee et al. demonstrated, with intracellular recordings of the transmembrane voltage in cortical cells, that the passive membrane parameters do not filter the extracellular AC fields at subthreshold membrane voltages (Lee et al., 2023). Thus, the decline in the PLV plots above 100 Hz (Figure 8) should not be because of passive filtering of high frequencies by the cell membrane at the PC level. Moreover, the PCs continued to be entrained at stimulation frequencies higher than their spontaneous firing rates by skipping one or multiple cycles of the AC (Asan et al., 2020). On the other hand, the time to the first spike following a synchronized PC inhibition in the large CN cells is about ~4 ms (see Figure 7 in Wu and Raman, 2017). For stimulation frequencies above 100 Hz, the jittering in the timing of the first spike following PC inhibition can be significant compared to the AC cycle length. Thus, the same level of jittering that does not have a significant effect on modulation at mid-frequencies can deteriorate the spike histograms and lower the PLV at higher frequencies. This limit at higher frequencies may simply be a reflection of the temporal precision at which the CN cells are capable of processing the information in PC spike synchrony patterns. The interesting finding is that the modulation can still be quite large at stimulation frequencies several times the cell’s spontaneous firing frequency by the mechanism of subharmonic modulation. In fact, it seems that the shortening of AC cycle length at higher frequencies increases the precision of PC synchrony until it is no longer useful in the face of biological jittering.

Finally, our results are in agreement with human trials where low frequency stimulation (10 Hz) of the cerebellum was ineffective and higher frequencies produced higher levels of modulation (Naro et al., 2016, 2017). Our entrainment plots show in general that the optimum range of frequencies for transsynaptic modulation is different in each CN cell but mostly above 50 Hz. However, it should be acknowledged that anesthesia can affect synaptic efficiencies and the preferred frequencies we show here for CN cells may shift in awake animals.

### 4.2. Electric field strengths

Transsynaptic, as opposed to direct (Tai and Tseng, 2022; Baker et al., 2023; Cajigas et al., 2023), modulation of deep cerebellar nuclei has the advantage that the injected current amplitudes can be much lower, and thus keep the electric fields within safe limits. In this study, for the largest stimulation current used (60 μA), the estimated electric field (150 mV/mm) at the cortical level was quite high compared to the e-fields intended (<1 mV/mm) at the targeted brain regions for clinical applications of transcranial stimulation (Guidetti et al., 2022). Modulation was detectable even at 10–20 μA in most cases, but our goal was to demonstrate a clear modulation of the cells above the noise floor to be able to look at the frequency tuning properties. Thus, we extended the upper range of the stimulus amplitude 2–3 times above the threshold levels. However, we believe that useful levels of CN modulation that can lead to functional benefits are possible at much lower electric fields when applied for longer periods of time.

Regarding the safety of the stimulation, as the AC frequency increases, the charge injected per half cycle decreases proportionally. In transcranial applications, the half-cell potential built-up on the surface electrodes will be reversed in the following half-cycle before it leads to chemical reactions with toxic byproducts. Similar arguments could be made in favor of AC stimulation regarding the maximum electric fields that can safely be induced on the excitable cells. Because of the transient nature of the AC currents, maximum allowable stimulation intensities may be much higher than DC stimulation, especially at high frequencies of AC, before harmful cellular effects can occur. Therefore, the safety limits on the currents injected and the electric fields induced at the cortical level should be evaluated on different grounds for transcranial DC and AC applications.

Although the direct effects on the CN cells through volume conduction were much smaller (~1.5 mV/mm at 60 μA) than those on the cortical cells, we cannot completely rule out the possibility of direct e-field effects on CN cells. But we conclude that the transsynaptic effect is the dominant mode of modulation in our data because of the following reasons: (1) The peak of the spike histograms are delayed with respect to the AC stimulus peaks by time intervals that agree with PC-CN propagation delay. (2) CN modulation levels are much lower at 10 Hz than higher frequencies and virtually absent at 1 Hz, 60 μA (data not shown). This strongly argues that the observed entrainment was transsynaptic because the e-fields have much longer time windows at lower frequencies to perturb the transmembrane potential if the effect was direct. (3) The SS synchrony mechanism for transsynaptic entrainment of CN cells predicts that low frequencies will be less effective as discussed above.

### 4.3. Cerebellar conductivity

Finally, the measured conductivity of the cerebellum (0.42 S/m) was slightly lower than 0.57 S/m that was predicted for the rat brain over the sensorimotor cortex (Asan et al., 2018), but still higher than some of the reported conductivities measured in the molecular and granular layers in the cat (0.17–0.3 S/m) (Yedlin et al., 1974) and turtle (0.15–0.25 S/m) cerebellar cortices (Okada et al., 1994). The higher cerebellar conductivity measured in our case may be explained by the contribution made by the underlying white matter that is highly anisotropic.

## 5. Conclusion

The cerebellar nuclear cells can be entrained through modulation of the cortical cells transsynaptically using AC stimulation. The CN cells have different ranges of stimulation frequencies for optimum entrainment and with peaks ranging between 50 Hz and 250 Hz. The 10 Hz AC stimulation, recently tested in human trials, is ineffective for transsynaptic modulation in the cerebellum. The overall interpretation of the data lead us to the conclusion that PC-SS synchrony is the most likely mechanism for the transsynaptic entrainment of the CN cells with AC stimulation of the cerebellar cortex. Indirect modulation of the CN by transcranial stimulation of the cerebellar cortex may be developed as an alternative method in clinical disorders where deep cerebellar stimulation is implicated as a treatment.

## Data availability statement

The original contributions presented in the study are included in the article/supplementary material, further inquiries can be directed to the corresponding author.

## Ethics statement

The animal study was approved by Institutional Animal Care and Use Committee, Rutgers University. The study was conducted in accordance with the local legislation and institutional requirements.

## Author contributions

QK: Writing – original draft, Formal analysis. EL: Writing – review & editing. MS: Writing – original draft, Writing – review & editing.
